# Delayed myocardial enhancement in pediatric hypertrophic cardiomyopathy: correlation with LV functional and demographic parameters

**DOI:** 10.1186/1532-429X-18-S1-P289

**Published:** 2016-01-27

**Authors:** Noha Behairy, Wesam E El Mozy, Sonia A EL Saiedi

**Affiliations:** 1 Radiology, Cairo University, Cairo, Egypt; 2 Pediatric, Cairo University, Cairo, Egypt

## Background

Hypertrophic cardiomyopathy is an important cause of sudden cardiac death throughout life and progressive heart failure. There are limited data on the prognostic significance of fibrosis in patients with HCM specially in the pediatric age group.

Our aim was to detect the presence of fibrosis in the pediatric age group and study its correlation with other demographic and LV functional parameter to detect additional risk stratification.

## Methods

We studied 30 pediatric patients diagnosed as HCM by echocardiography. All patients were submitted to clinical examination in which the NYHA classification was determined for each patient, echocardiography and CMR. 2D echocardiography was done with measuring the pressure gradient across the LVOT. CMR was done on a 1.5T Philips Achieva scanner in SSFP with delayed myocardial enhancement. Images were analyzed on the Philips workstation for functional parameters and on Diagnosoft program for percentage of myocardial enhancement. All demographic and functional parameters as well as pressure gradient were correlated with the percentage of myocardial enhancement. Written approval was obtained from the patients guardian as well as the approval of the local ethical committee.

## Results

We studied 11 female and 19 male patients from 45 days up to 18yrs. Eight patients showed positive family history for HCM. The mean for percentage of myocardial enhancement was 9.7 ± 9. Only 5 patients didn't show enhancement. We found significant correlation between the NYHA classification and the pressure gradient across the LVOT (P = <0.001) as well as the percentage of myocardial enhancement (P = 0.004). The percentage of myocardial enhancement showed positive correlation with LV myocardial mass index (p = 0.042). It didn't correlate with any other demographic or LV functional cardiac parameters . A good positive correlation was detected between the percentage of myocardial enhancement and the severity of pressure gradient measured by echocardiography (r = 0.69 and P=<0.001).

## Conclusions

We found a significant correlation between the percentage of myocardial enhancement in pediatric HCM and the pressure gradient, NYHA classification and LV myocardial mass . This may help in further management of those patients, planning of follow up and prognosis of the disease.

**Table 1:** LV MRI data for the patients

DE% (mean and SD)	9.7 +9
EF (median and range)	71 (38-88)
EDV (mean and SD)	63.9+ 40.9
ESV (median and range)	21.5 (3-95)
SV (mean and SD)	46.2+ 25.8
CO (median and range)	3.9 (2.4-8.9)
CI (mean and SD)	4.2+ 1.4
LV myocardial mass indexed (mean and SD)	108.02 ± 68

All measurements are indexed to the body surface area

**Figure 1:**
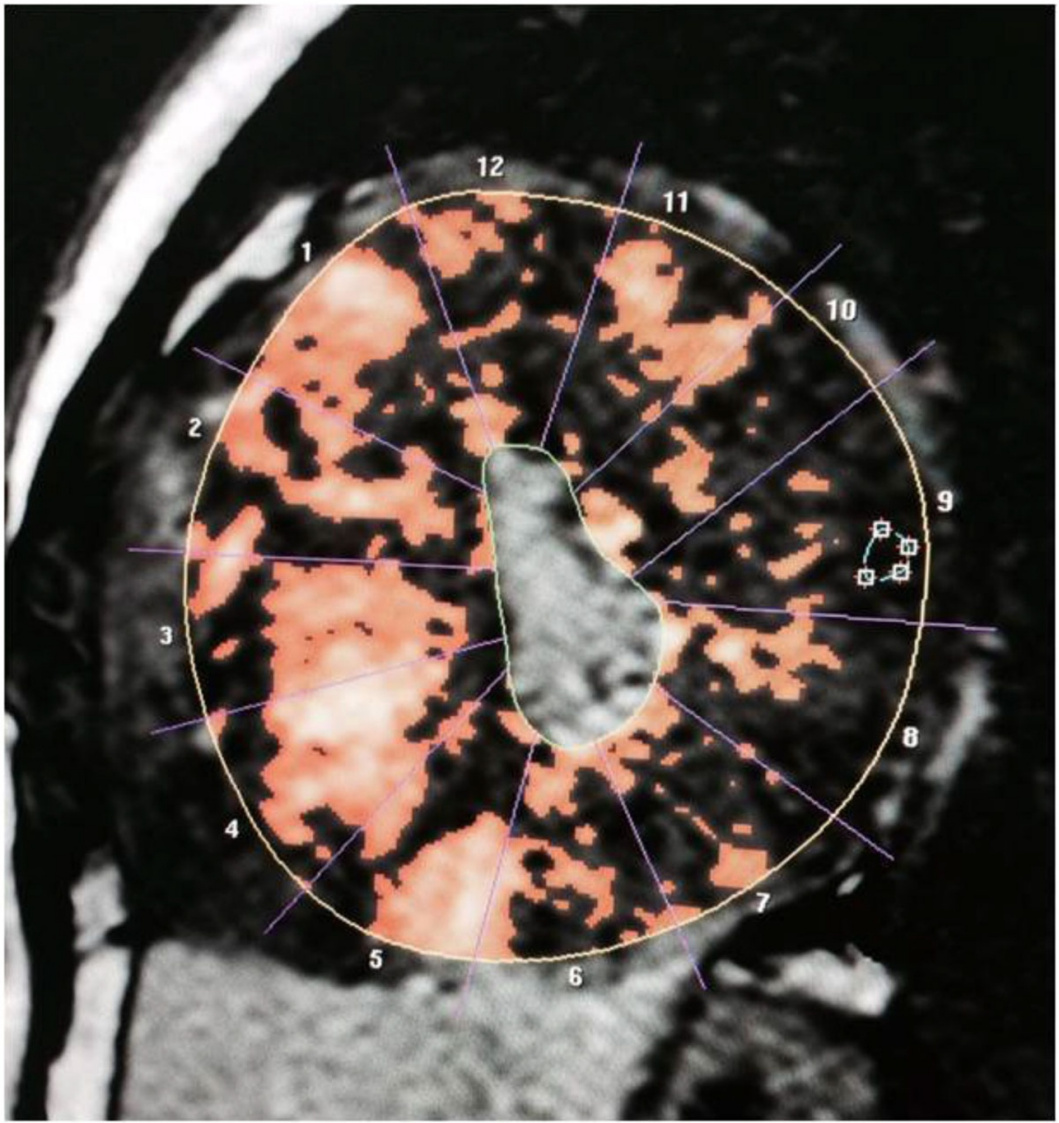
**Short axis image of an 18yr old patient with HCM shows concentric hypertrophy of the myocardial wall with extensive areas of myocardial enhancement**.

